# Mechanical Properties and Corrosion Behavior of Silica Nanoparticle Reinforced Magnesium Nanocomposite for Bio-Implant Application

**DOI:** 10.3390/ma15228164

**Published:** 2022-11-17

**Authors:** AKM Asif Iqbal, Norfatihah Binti Ismail

**Affiliations:** 1 Department of Mechanical, Materials and Manufacturing Engineering, University of Nottingham Ningbo China, Ningbo 315100, China; 2 Faculty of Manufacturing and Mechatronic Engineering Technology, University Malaysia Pahang (UMP), Pekan 26600, Pahang, Malaysia

**Keywords:** magnesium matrix composite, nanocomposite, biomaterial, nanosilica, mechanical properties, corrosion

## Abstract

In this study, magnesium (Mg)-based nanocomposites reinforced with silica (SiO_2_) nanoparticles were developed using the powder metallurgy process, and their mechanical and corrosion behavior were assessed. Mg-alloy AZ31 served as the matrix material, and two different weight percentages of SiO_2_ nanoparticles were used as filler. According to the microstructural analysis, the composite generated a Mg_2_Si phase as a result of SiO_2_ dissociating during the sintering process. The microhardness of the Mg-alloy dramatically enhanced with the addition of 3% nanosilica, although the elastic modulus remained constant. Additionally, the outcomes demonstrated that the Mg_2_Si phase’s development in the composite constrained the mechanism of deterioration and postponed the pace of degradation, which aided in enhancing the qualities of corrosion resistance. This nanocomposite might, thus, be thought of as a potential replacement for the traditional bio-implant materials.

## 1. Introduction

Traditional metals such as titanium, stainless steel, and platinum have great strength, corrosion-resistance, and biocompatibility properties; as a result, they are frequently employed in orthopedic implants used in bone fracture surgery [1]. The implants made from these materials are generally present in the body, even after the mending of the damaged tissue has caused infection due to the implant material’s corrosion under physiological conditions. However, these materials are not biodegradable. Another drawback of these metallic materials is their high elastic moduli that leads to stress-shielding effect [1]. Due to this, removing the implant typically requires a revision operation, which is quite inconvenient for the patients [2]. Therefore, creating a cutting-edge biodegradable implant material that can preserve the mechanical qualities of the bones becomes of utmost importance.

In recent years, magnesium (Mg) and its alloys have generated a great deal of interest as prospective substitutes for standard orthopedic implant materials due to their good mechanical and biodegradable qualities [3,4,5]. With a density range of 1.74 to 2.0 gm/cc and a fantastic strength-to-weight ratio, this is the lightest metal [6]. Additionally, the elastic modulus of magnesium alloys, which ranges between 41 and 45 GPa and is comparable to that of cortical bone, would lessen the likelihood of the stress shielding effect [7]. More importantly, these materials are biodegradable and, hence, completely absorbed in the human body after regeneration of the bone tissue [8]. Despite their great advantages, these Mg materials show poor corrosion resistance in a physiological environment [9]. The remarkably high rate of disintegration in contact with bodily fluid prevents their application in the creation of bio-implants. Composites made of Mg alloys with filler materials added have better corrosion behavior and preserve deterioration at a regulated pace. The corrosion and mechanical characteristics of a magnesium matrix composite may be altered by choosing reinforcing elements with varied content, distribution, and size. Numerous reinforcing materials have been used in this respect, including hydroxyapatite (HAP) [10], zinc oxide [11], bioactive glass (BG) [12], calcium particles [13], calcium polyphosphate particles (CPP), and calcium phosphate-based ceramics [14,15]. In addition to these materials, many oxide materials have been employed as fillers to create Mg-based composites, including alumina (Al_2_O_3_) [16], titania (TiO_2_) [17], zirconia (ZrO_2_) [18], and silica (SiO_2_) [19]. To enhance the corrosion behavior of the AZ91D magnesium alloy, Amiri et al. [18] added ZrO_2_ coating. Due to its lack of biocompatibility, this material, while having superior degradation resistance, has not been widely used [20]. Additionally, Al_2_O_3_ fillers were employed by Kang et al. [16] to create magnesium scaffolds for bio-implant applications. They discovered that the corrosion rate in the composite with 5 wt% Al_2_O_3_ was higher than that of pure magnesium in SBF solution. As a result, they applied a MgF_2_ coating, which significantly reduced the rate of corrosion. TiO_2_ has also been used to coat magnesium by Amravati et al. [17], although its application as a reinforcing filler is quite restricted. Due to its excellent biocompatibility, SiO_2_ was used in the development of Mg-based composite for various biomedical applications in addition to the aforementioned oxide materials [21]. It aids in bone repair and regeneration and is bioactive, biodegradable, and non-toxic in human bodily fluids [22,23]. Moreover, silica and silica compounds demonstrate inherent anti-corrosive properties [24]. During the manufacture of magnesium-based composites, SiO_2_ produces the intermetallic complex magnesium silicide (Mg_2_Si), which has a substantial impact on the materials’ mechanical and corrosion characteristics. Few studies have examined Mg_2_Si production in Mg-based composites and its effects on corrosion and mechanical properties. Compared to pure magnesium, the Mg–SiO_2_ composite made by mechanical alloying has enhanced fracture toughness, according to Wang et al. [25]. The Mg_2_Si that the composite created had a significant impact on fracture toughness. The Mg_2_Si intermetallic compounds were similarly discovered by Kondoh et al. [26] when utilizing comparable materials and repeatedly performing compaction and extrusion. Additionally, Lu et al. [27] treated the elemental Mg and Si powders using mechanical alloying and looked into the formation of Mg_2_Si and its beneficial effects on enhancing the mechanical characteristics of the composite. When Sun et al. [28] examined the Mg–Mg_2_Si composite’s production kinetics, they discovered that the elemental powders remained inert below 580 °C. Further, Myalska et al. [29] created in-situ Mg-based composites using hydrophilic fumed silica nanoparticles. Their findings suggested that the formation of Mg_2_Si and MgO on the composite had an impact on its mechanical characteristics. Ben-Hamu et al. [30,31] noted the occurrence of Mg_2_Si intermetallic in the wrought Mg–Zn–Mn alloy and came to the conclusion that the Mg_2_Si boosted the alloy’s corrosion resistance. Although the aforementioned study shows that SiO_2_ has an impact on Mg-based composites, its impact on the ability of Mg alloy AZ31 to dissociate into Mg_2_Si has not been investigated. Therefore, the goal of this project is to create a SiO_2_ nanoparticle reinforced Mg alloy AZ31 composite and assess its mechanical and corrosion characteristics in order to use it as a possible bio-implant material.

## 2. Materials and Methods

**Figure 1:**
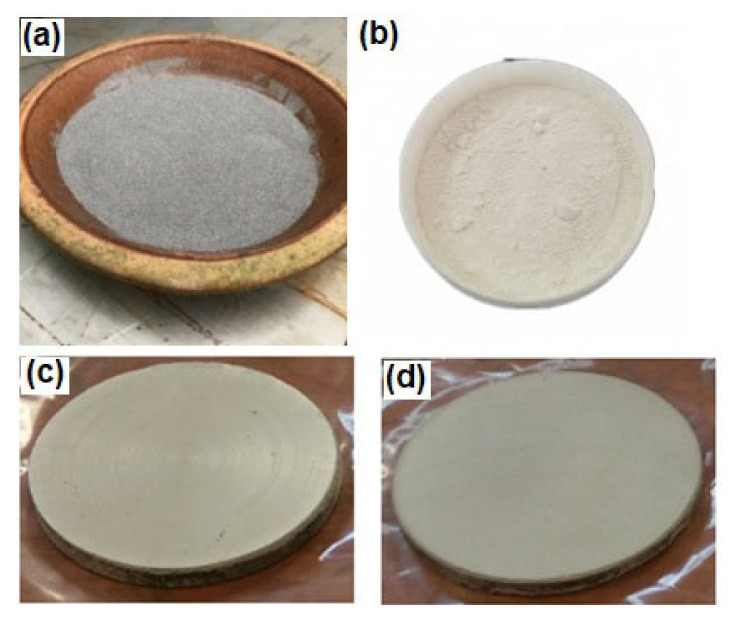
(**a**) Magnesium powder, (**b**) SiO_2_ nanopowder, (**c**,**d**) composite samples after sintering.

Silica (SiO_2_) nanopowder of 99.9% purity with an average particle size of 35 nm was utilized as the reinforcement and magnesium alloy AZ31 powder with an average particle size of 56 µm was used as the matrix material (bought from Sigma Aldrich, Malaysia). Table 1 displays the chemical composition of Mg-alloy AZ31, and Table 2 displays the mechanical characteristics of the matrix and reinforcing components. The composite was made using the traditional powder metallurgy method. In this experiment, two different weight percentages of SiO_2_ reinforcement, 3 wt% and 5 wt% were utilized, and the composites that were produced were compared to the base material. In order to achieve homogeneous mixing, pure Mg powder and SiO_2_ nanopowder were initially combined in a planetary ball mill (RETSCH PM 100(Haan, Germany) for 1 h at a rotating speed of 150 rpm. As a process control agent, polyvinyl alcohol (PVA) was included into the powder combination. The PVA additive prevents the powder from adhering to the walls of the vial and the balls, which reduces agglomeration and enhances mixing quality. The powder mixture was heated in an oven to 100 °C for one hour to evaporate the combination’s volatile components. After that, the powder combination was compressed. For the purpose of compaction, a uniaxial hydraulic press (TOYO: TL30 (TOYO Electric Corporation, Aichi, Japan), 300 kN capacity) was employed. Green compacts with a diameter of 40 mm and a thickness of 3 mm were produced when 200 kN of compaction force was applied. The samples were then sintered for one hour at 570 °C in a muffle furnace. An ongoing argon gas flow was kept throughout the sintering procedure to prevent oxidation. Finally, Mg nanocomposites with SiO_2_ reinforcement were developed and ready for various tests. The raw powders and the sintered sample of composites are shown in Figure 1. All of the samples were sequentially polished with various abrasive paper grades, followed by successive polishing with diamond suspension of 10 μm, 5 μm, and 1 μm to obtain mirror polish, in order to study the microstructure of the produced composites. The sintered samples were optically photographed using a metallurgical microscope (OLYMPUS BX51M, Tokyo, Japan). The microstructure of the manufactured nanocomposite samples was examined using an energy dispersive spectrometer (EDS) and a scanning electron microscope (SEM JEOL 6390, Tokyo, Japan). The phase formation of the sintered samples was observed by X-ray diffraction (XRD). The XRD of the fabricated nanocomposite was performed using a high-resolution X-ray diffractometer (Shimadzu XRD 6000 (Shimadzu Corporation, Kyoto, Japan)) with a voltage of 45 kV and a tube current of 40 mA. Additionally, a helium pycnometer was used to gauge the density of all the manufactured samples. The density of each sample was determined by testing it five times and taking the average. All of the manufactured samples’ micro-hardness was also assessed. The micro-indentation tests were performed on the polished surface of the samples using Vickers hardness tester (Wilson Hardness: Model 402 MVD, (Wilson Instruments, Norwood, MA, USA). The tests were carried out at room temperature according to the instruction of ASTM E384. In this experiment, the sample was loaded with 0.7 N at a loading rate of 1 N/min, and a dwell duration of 5 s was maintained during the whole indentation. Ten repeated measurements were made on each surface at intervals of 1 mm to exclude the impact of the nearby indentations. The mean value was taken as the Vickers hardness (HV) value. The load–displacement curve obtained from the experiment was used to determine the microhardness and the elastic modulus by using the Oliver–Pharr method. 

The electrochemical corrosion assessment of the manufactured samples was carried out in a 37 °C Phosphate Buffer Saline (PBS) solution. A three-electrode cell with a platinum counter electrode, a saturated calomel electrode serving as a reference electrode, and a test sample serving as a working electrode was used to conduct the experiments. The scan rate used for the investigations was 0.5 mVs^−1^. Before the electrochemical test, all of the samples were polished and cleaned with acetone. SEM and EDX investigations were carried out after the test to examine the microstructure of the corroded samples.

## 3. Results and Discussion

### 3.1. Microstructural Characterization

**Figure 2:**
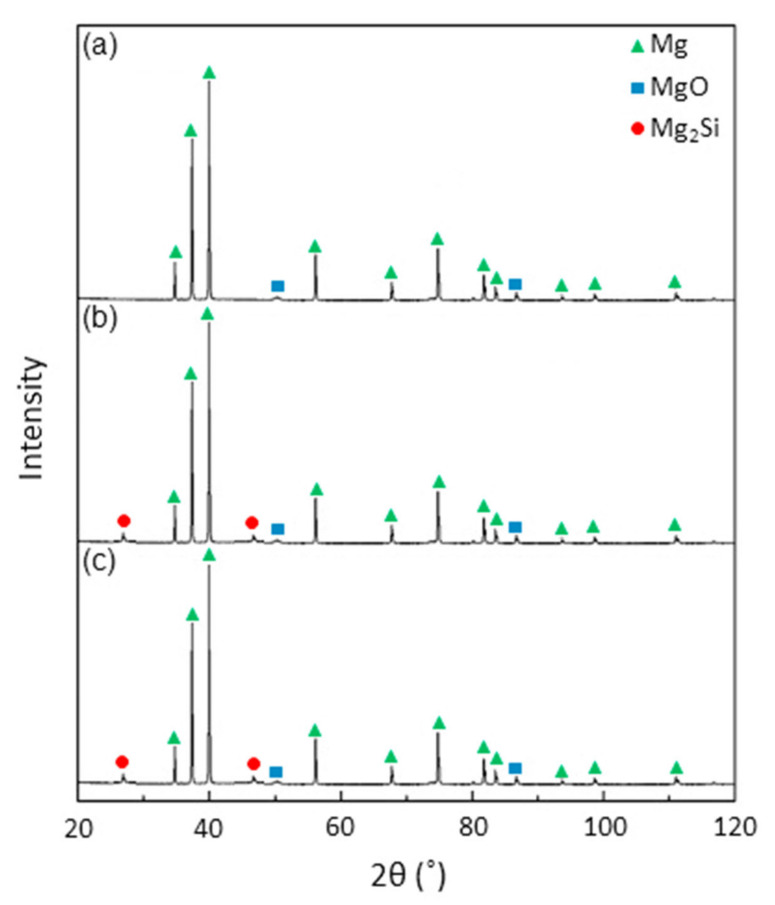
XRD pattern of sintered (**a**) Mg alloy AZ31, (**b**) Mg + 3% SiO_2_, (**c**) Mg + 5% SiO_2_.

**Figure 3:**
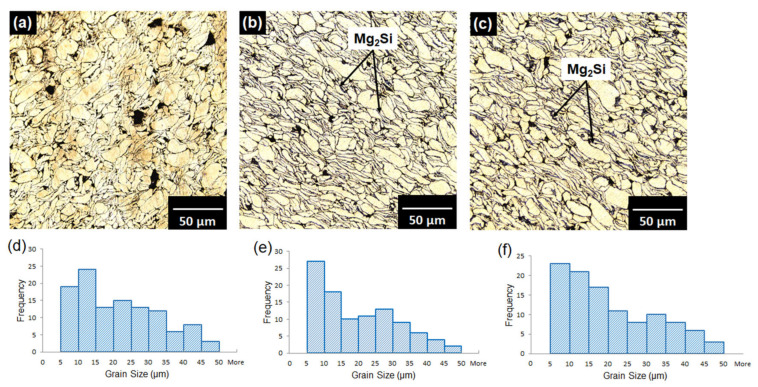
Optical micrograph and grain size histogram of (**a**,**d**) Mg alloy AZ31, (**b**,**e**) Mg + 3% SiO_2_, (**c**,**f**) Mg + 5% SiO_2_.

**Figure 4:**
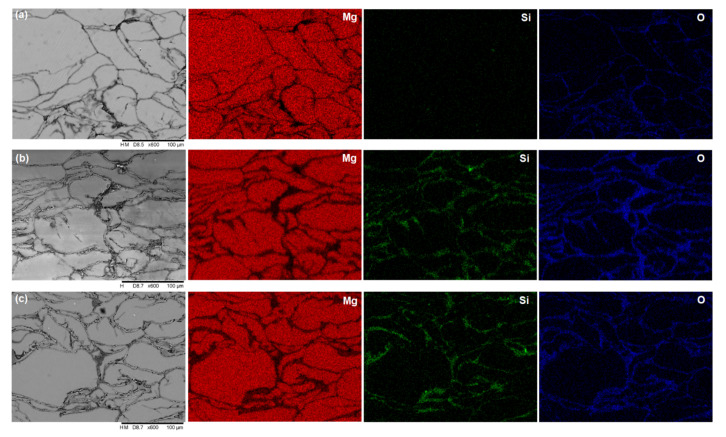
SEM micrograph and EDX elemental mapping of (**a**) Mg alloy, (**b**) Mg + 3% SiO_2_, (**c**) Mg + 5% SiO_2_.

The XRD patterns of pure magnesium and Mg–SiO_2_ nanocomposites with two distinct weight percentages of silica are shown in Figure 2. The strongest pyramidal peak is seen in all samples at 40.1°, followed by the basal peak at 37.1° and the prismatic peak at 34.9°. These three peak locations in the samples show that the addition of SiO_2_ nanoparticle had no impact on the texture of magnesium. Moreover, Mg_2_Si peaks are seen at 27.6° and 47.3° in both composite samples. Additionally, all of the samples exhibit MgO peaks at 50.9° and 87.2°. As a result, the XRD data unambiguously show that Mg_2_Si is present in the nanocomposites created during the sintering process. Figure 3 displays the optical micrograph of the sintered samples and the related histogram of grain size. The grain boundary is indicated by the dark black lines in the optical micrographs. All of the samples’ optical micrographs reveal a finely sintered microstructure. Mg_2_Si is predicted to develop along the grain boundaries in both of the composite samples (Figure 3b,c), however due to the dark, black boundary lines, this is not apparent in the optical micrograph. Using ImageJ software, the grain size was determined, and histograms were created based on the findings from 300 grains. Mg, Mg + 3% SiO_2_, and Mg + 5% SiO_2_ have typical grain sizes of 21 µm, 18 µm, and 16 µm, respectively. This result exhibited that the grain size of Mg reduces once the silica nanoparticles are added to the Mg alloy. However, the grain size reduction in Mg is not so significant with the addition of 3% and 5% SiO_2_. SEM and EDX investigations were also carried out to observe the production of the Mg_2_Si compound. Figure 4 displays the SEM micrograph and relevant EDX maps of the sintered composite samples. The fact that no SiO_2_ nanoparticle reinforcement aggregated in the composite surface according to the SEM data shows that the fabrication process’s blending and sintering steps were carried out perfectly. The presence of Mg, Si, and O in the elemental concentration determined by SEM and EDX analysis points to the occurrence of MgO and Mg_2_Si phases at the particle boundaries. This finding demonstrates unequivocally that after sintering, a layer of Mg_2_Si is created in the composite, which may have an impact on the mechanical and corrosion characteristics of the composite. 

### 3.2. Mechanical Characterization

**Figure 5:**
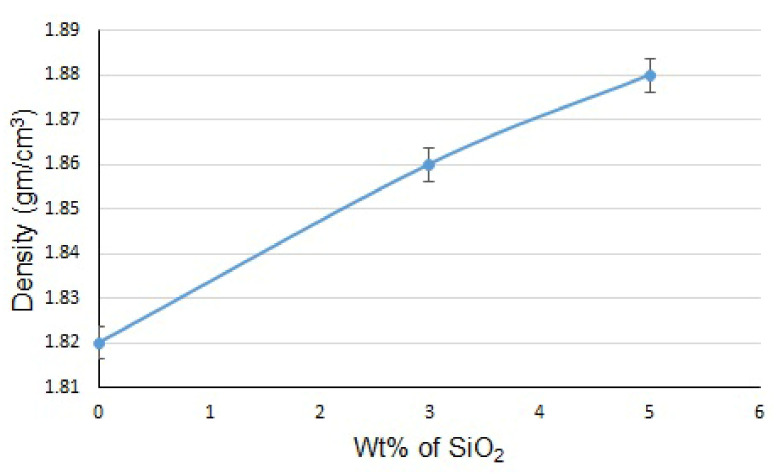
Density of Mg-based composite with different wt% of SiO_2_.

The density of the Mg alloy AZ31 and the composites with 3 and 5 wt% SiO_2_ are shown in Figure 5. Mg alloy AZ31 has an experimental density of 1.81 gm/cm^3^, which is a little bit higher than the theoretical density of 1.8 gm/cm^3^. From the figure, it can be seen that the density of both composites is higher than that of the base material. Mg + 3% SiO_2_ and Mg + 5% SiO_2_ have respective densities of 1.86 gm/cm^3^ and 1.88 gm/cm^3^, which are 2.7% and 3.8% greater than the base material. The addition of SiO_2_ in the Mg alloy develops MgO and Mg_2_Si phases in the composite which possess a relatively high theoretical density of 3.58 gm/cm^3^ and 1.99 gm/cm^3^, respectively, than that of Mg alloy.

**Figure 6:**
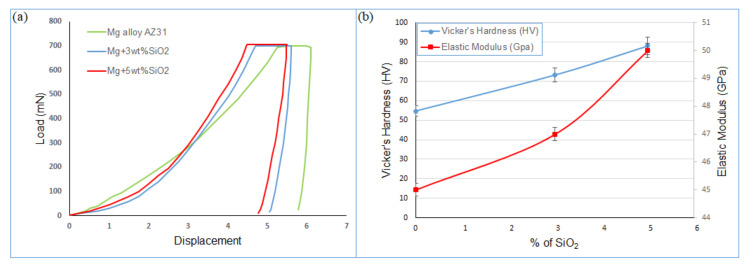
(**a**) Load–displacement curve, (**b**) microhardness and elastic modulus of composites with different wt% of SiO_2_.

As a result, the total density of the sintered composites is increased by the inclusion of these two phases. Additionally, the sintered samples’ microhardness has been examined. The load–displacement curve obtained after the microhardness test is shown in Figure 6a, and the elastic modulus and microhardness results of the Mg alloy, Mg + 3% SiO_2_, and Mg + 5% SiO_2_ samples are shown in Figure 6b. The elastic modulus was calculated based on the load–displacement curve as shown in Figure 6a. Both composites exhibit higher hardness values than the base metal (Figure 6b). The microhardness of the Mg + 3% SiO_2_ and Mg + 5% SiO_2_ composites were higher than those of the Mg matrix alloy by 33.8% and 61%, respectively. This higher value of hardness in the composite samples is attributed to the existence of Mg_2_Si. The addition of nanosilica reacts with Mg and produces Mg_2_Si intermetallic phase which possesses a high hardness value results to increase the overall hardness of the composites. Moreover, Figure 5b demonstrates the elastic modulus of the composites calculated from the load–displacement curve obtained after the microhardness test. The elastic modulus values of the Mg + 3% SiO_2_ and Mg + 5% SiO_2_ composites are 47 GPa and 51 GPa, respectively, which are quite similar to the 45 GPa elastic modulus value of the Mg alloy. This shows that the inclusion of SiO_2_ nanoparticles does not appreciably alter the elastic modulus of Mg alloy. This outcome rather suggests the potential of the fabricated composites for usage as a bio-implant material. One of the crucial mechanical characteristics that must be taken into account for the biomaterial aiming for orthopedic implant applications is elastic modulus. The elastic modulus of cortical bone is typically 10–20 GPa [32]. The elastic modulus of the titanium and stainless-steel implant materials that are commercially accessible ranges from 100 to 200 GPa [32]. A decrease in bone mass, commonly known as bone resorption, is caused by the significant elastic modulus difference between cortical bone and typical implants. This restriction necessitates revision surgery in order to remove the implant material. The manufactured Mg + SiO_2_ nanocomposites in this work do not exhibit a significant variation in stiffness from natural bone. Moreover, Mg’s guaranteed biodegradability negates the necessity for corrective surgery. As a result, the created SiO_2_ reinforced Mg nanocomposite may one day serve as a cheaper alternative to the current high-end traditional implants. 

### 3.3. Corrosion Behavior

**Figure 7:**
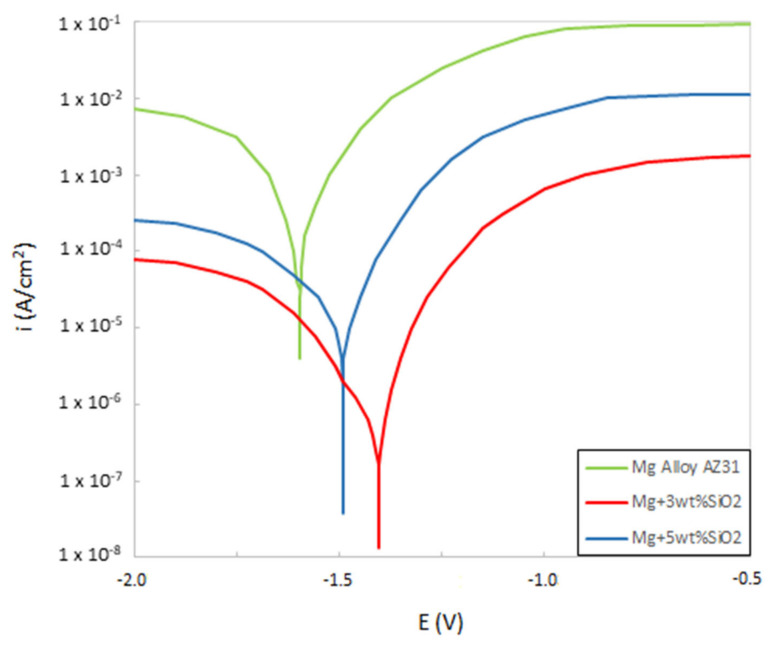
Potentiodynamic curves of Mg alloyAZ31, Mg + 3 wt% SiO_2_ and Mg + 5 wt% SiO_2_ composites.

**Figure 8:**
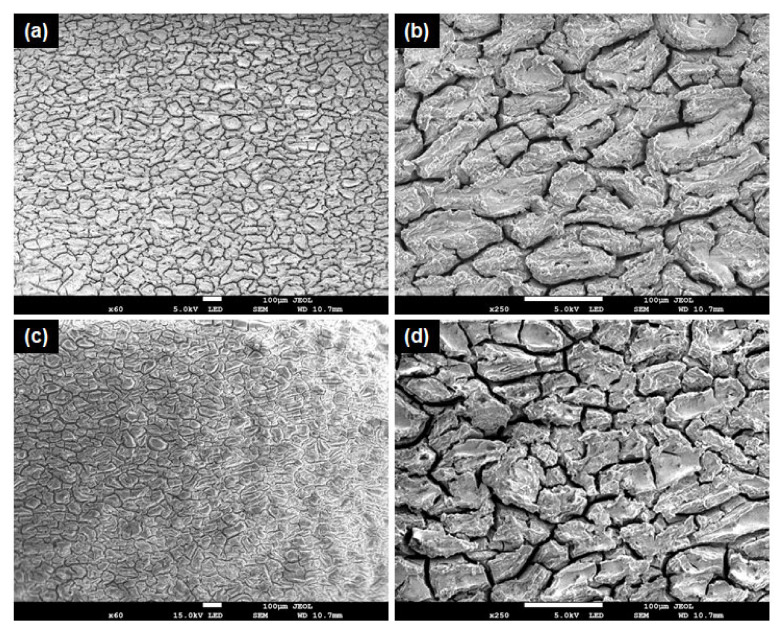
SEM micrograph of the corroded samples (**a**,**b**) Mg Alloy, (**c**,**d**) Mg + 3% SiO_2_.

**Figure 9:**
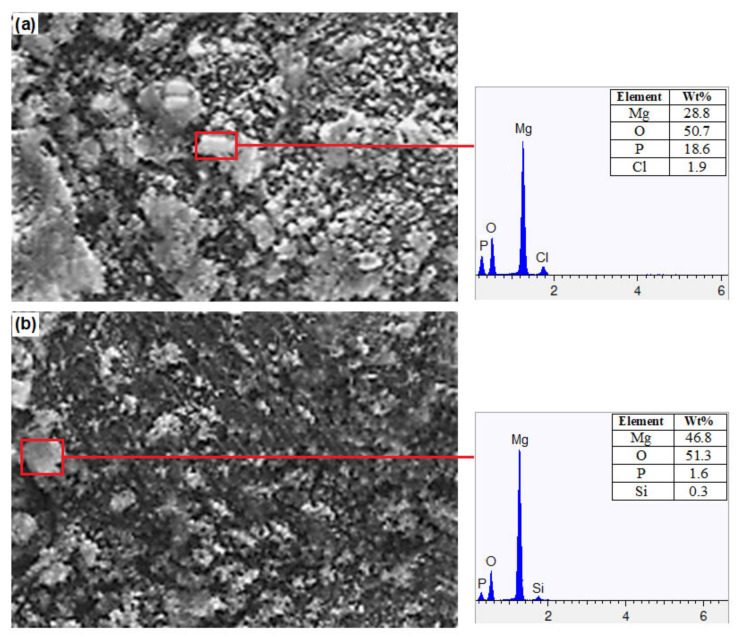
EDX analysis of the corroded samples (**a**) Mg Alloy, (**b**) Mg + 3% SiO_2_.

Figure 7 shows the potentiodynamic polarization curves for Mg alloy, Mg + 3 wt% SiO_2_, and Mg + 5 wt% SiO_2_. It should be noted that when SiO_2_ is added to Mg alloy, corrosion potential (Ecorr) increases and corrosion current density (Icorr) falls in comparison to the base alloy, showing that the composites’ corrosion resistance has been improved. However, compared to the other composite, the one with 3 wt% SiO_2_ exhibits superior corrosion resistance. This states that the corrosion resistance characteristic degrades when SiO_2_ is added in amounts more than 3 weight percent. Additionally, it was determined that the corrosion rates of Mg + 3% SiO_2_ and Mg + 5% SiO_2_ were 0.3 mm/y and 0.56 mm/y, respectively, which is very slow when compared to the rate of 39.7 mm/y for Mg alloy AZ31. Therefore, it is clear that the addition of SiO_2_ to the Mg alloy can improve the corrosion resistance and corrosion rate significantly. Furthermore, SEM and EDX examination were carried out to investigate the corrosion surface state. Figure 8 depicts the SEM micrograph of the corroded Mg-alloy surface and the Mg + 3 wt% SiO_2_ composite surface. The degradation layer (whitish-gray part in Figure 8) was observed spread over the whole surface in the Mg alloy and Mg + 3% SiO_2_ composite. However, Mg + 3% SiO_2_ composite becomes less corroded in comparison to the Mg alloy. Figure 9 exhibits EDX analysis of Mg alloy and Mg + 3 wt% SiO_2_. The corroded portion of the Mg alloy contains Mg, Phosphorus (P), Oxygen (O), and Chlorine (Cl). This means that Mg(OH)_2_ and MgCl_2_ are formed as the corrosion product in the Mg alloy that accelerates the corrosion in the Mg alloy. The Mg + SiO_2_ sample, on the other hand, shows the presence of Mg, O, P, and Silicon (Si), but Cl was not present, indicating that no MgCl_2_ was generated on the corroded portion of the Mg + 3 wt% SiO_2_ composite. The Mg_2_Si phase that evolved in the composite during the sintering process inhibits Cl ion and stabilizes the hydroxide coating created during corrosion and functions as a preventative barrier to the corrosion since silica materials naturally have anti-corrosive properties [24]. Consequently, this aids greatly in improving corrosion resistance. Hence, the nanocomposite fabricated with 3 wt% SiO_2_ could be a viable candidate as a biomaterial that can delay the degradation process of the implant in a human body.

## 4. Conclusions

In this study, SiO_2_ nanoparticle reinforced Mg-based composites have been developed by using powder metallurgy technique. Through this technique, a uniform distribution of reinforcement was attained, and a Mg_2_Si intermetallic phase evolved in the composite, which had an impact on the composites’ mechanical and corrosion properties. The composite samples performed better in mechanical tests in terms of density and hardness, but their elastic modulus was identical to that of the underlying Mg alloy. This indicates the compatibility of the nanocomposite with the cortical bone in terms of mechanical properties. The composite samples made with 3 wt% SiO_2_ showed better corrosion resistance properties in the potentiodynamic tests. The Mg_2_Si phase formed during the fabrication process restricted the formation of hydroxide and delayed the degradation process. Hence, it can be concluded that the fabricated Mg + 3 wt% SiO_2_ could be a potential candidate to replace the conventional biomaterial for implants and fixation devices. 

## Figures and Tables

**Table 1:** Chemical composition of magnesium alloy AZ31 (wt%).

Al	Zn	Mn	Cu	Mg
2.83	0.8	0.37	0.002	Balance

**Table 2:** Mechanical properties of matrix and reinforcement materials.

Parameter	Mg Alloy AZ31	SiO_2_
Density (g/cm^3^)	1.8	2.6
Tensile Strength (MPa)	290	135
Young’s Modulus (GPa)	45	70
Poisson’s ratio	0.35	0.19

## Data Availability

Not applicable.
